# The Use of ChatGPT for Education Modules on Integrated Pharmacotherapy of Infectious Disease: Educators' Perspectives

**DOI:** 10.2196/47339

**Published:** 2024-01-12

**Authors:** Yaser Mohammed Al-Worafi, Khang Wen Goh, Andi Hermansyah, Ching Siang Tan, Long Chiau Ming

**Affiliations:** 1 College of Medical Sciences Azal University for Human Development Sana’a Yemen; 2 College of Pharmacy University of Science and Technology of Fujairah Fujairah United Arab Emirates; 3 Faculty of Data Science and Information Technology INTI International University Nilai Malaysia; 4 Department of Pharmacy Practice Faculty of Pharmacy Universitas Airlangga Surabaya Indonesia; 5 School of Pharmacy KPJ Healthcare University Nilai Malaysia; 6 School of Medical and Life Sciences Sunway University Selangor Malaysia

**Keywords:** innovation and technology, quality education, sustainable communities, innovation and infrastructure, partnerships for the goals, sustainable education, social justice, ChatGPT, artificial intelligence, feasibility

## Abstract

**Background:**

Artificial Intelligence (AI) plays an important role in many fields, including medical education, practice, and research. Many medical educators started using ChatGPT at the end of 2022 for many purposes.

**Objective:**

The aim of this study was to explore the potential uses, benefits, and risks of using ChatGPT in education modules on integrated pharmacotherapy of infectious disease.

**Methods:**

A content analysis was conducted to investigate the applications of ChatGPT in education modules on integrated pharmacotherapy of infectious disease. Questions pertaining to curriculum development, syllabus design, lecture note preparation, and examination construction were posed during data collection. Three experienced professors rated the appropriateness and precision of the answers provided by ChatGPT. The consensus rating was considered. The professors also discussed the prospective applications, benefits, and risks of ChatGPT in this educational setting.

**Results:**

ChatGPT demonstrated the ability to contribute to various aspects of curriculum design, with ratings ranging from 50% to 92% for appropriateness and accuracy. However, there were limitations and risks associated with its use, including incomplete syllabi, the absence of essential learning objectives, and the inability to design valid questionnaires and qualitative studies. It was suggested that educators use ChatGPT as a resource rather than relying primarily on its output. There are recommendations for effectively incorporating ChatGPT into the curriculum of the education modules on integrated pharmacotherapy of infectious disease.

**Conclusions:**

Medical and health sciences educators can use ChatGPT as a guide in many aspects related to the development of the curriculum of the education modules on integrated pharmacotherapy of infectious disease, syllabus design, lecture notes preparation, and examination preparation with caution.

## Introduction

Artificial intelligence (AI) plays an important role nowadays rather than at any time in history in many fields, including medical education, practice, and research [1-6]. AI can be defined as the “science and engineering of making intelligent machines, especially intelligent computer programs. It is related to the similar task of using computers to understand human intelligence, but AI does not have to confine itself to methods that are biologically observable” [7], or as “a field of science and engineering concerned with the computational understanding of what is commonly called intelligent behaviour, and with the creation of artefacts that exhibit such behaviour” [8]. One of the recent advances in AI development is the launch of a model called ChatGPT, which interacts in a conversational way. The dialogue format makes it possible for ChatGPT to answer follow-up questions, admit its mistakes, challenge incorrect premises, and reject inappropriate requests; ChatGPT is a general large language model (LLM) developed recently by OpenAI. While the previous class of AI models have primarily been deep learning models, which are designed to learn and recognize patterns in data, LLMs are a new type of AI algorithm trained to predict the likelihood of a given sequence of words on the basis of the context of the words that appear before it [9].

Empirical studies have demonstrated the effectiveness of AI-based educational tools in various domains. Recent research published in *JMIR Medical Education* [10] on February 8, 2023, evaluated ChatGPT's potential as a medical education instrument. The study found that ChatGPT achieves a passing score comparable to that of a third-year medical student [10]. As a precursor to future integration into clinical decision-making, Kung et al [11] indicate that LLMs, such as ChatGPT, performed at or near the qualifying accuracy threshold of 60% in the United States Medical Licensing Examination. Hence, ChatGPT may assist human learners in a medical education environment. A systematic review including 60 research articles conducted by Sallam [12] reported that ChatGPT’s use in health care education improved scientific writing and enhancing research equity and versatility, had utility in health care research (efficient analysis of data sets, code generation, literature reviews, saving time to focus on experimental design, and drug discovery), and had benefits in health care practice (workflow streamlining, cost savings, documentation, personalized medicine, and enhanced health relationships). Many educators, researchers, health care professionals and students started using ChatGPT at the end of 2022 for many purposes, such as preparing lecture notes, assignments, literature reviews, and others. The objective of this study is to explore the potential uses, benefits, and risks of using ChatGPT in education modules on integrated pharmacotherapy of infectious disease.

## Methods

### Study Design

A content analysis of the potential applications of the ChatGPT model for education modules on integrated pharmacotherapy of infectious disease was performed. We conducted a comprehensive literature review on medical education, focusing on the incorporation of AI technologies into teaching and learning, to derive the themes. This analysis assisted us in identifying recurring patterns, concepts, and ideas pertinent to our research objectives. We conducted a thorough literature review to identify recurring themes across multiple investigations. These themes served as the basis for our discussion and analysis. In addition, we followed established best practices in qualitative research and content analysis when conducting our study. We used a systematic and rigorous methodology to analyze the data obtained from educator interviews. Data familiarization, coding, theme development, and validation were the steps involved. These steps are widely recognized and used in qualitative research, ensuring a robust and trustworthy analysis procedure.

Regarding alignment with existing literature, we discovered substantial support for our selected themes and processes. Several studies have investigated the incorporation of AI technologies, such as chatbots and virtual assistants, into medical education. Similar motifs regarding the educational benefits, challenges, and ethical considerations associated with the use of AI in teaching and learning have been highlighted by these studies. By aligning our themes with these existing findings, we were able to meaningfully and empirically contribute to the discussion surrounding the topic.

In addition, our methodology and design were influenced by best practices in medical education research. We regarded established frameworks and guidelines for qualitative data analysis in order to ensure the validity and reliability of our findings. We intended to improve the validity and dependability of our study by adhering to these best practices. Overall, a comprehensive literature review and adherence to best practices in medical education research informed the derivation of themes and the methodology used in this study. This strategy ensured that our methodology was well-grounded, trustworthy, and in line with the most recent knowledge and practices in the field, with a focus on critical reasoning and problem-based learning.

### Data Collection

#### Overview

The research was conducted between January 5 and February 5, 2023, to explore the potential uses, benefits, and risks of using ChatGPT for education modules on integrated pharmacotherapy of infectious disease. Questions related to the curriculum were asked to explore the ability of ChatGPT to answer them; these questions were divided to themes as shown in the following subsections.

#### Theme 1

Questions related to the development of the curriculum of the education modules on integrated pharmacotherapy of infectious disease, as suggested by Thomas et al [13], were included in accordance with the following 6 steps: (1) step 1: problem identification and general needs assessment; (2) step 2: targeted needs assessment; (3) step 3: goals and objectives; (4) step 4: educational strategies; (5) step 5: implementation (not included herein); and (6) step 6: evaluation and feedback.

#### Theme 2

Questions related to the syllabus for each topic, such as integrated pharmacotherapy of respiratory tract infections, were included.

#### Theme 3

Questions related to the preparation of lecture notes related to each topic, such as integrated pharmacotherapy of respiratory tract infections, were included.

#### Theme 4

Questions related to the preparation of examinations with model answers related to each topic, such as integrated pharmacotherapy of respiratory tract infections, were included.

### Data Analysis

The performance of the ChatGPT model in providing answers for the education modules on integrated pharmacotherapy of infectious disease was extensively assessed. To ensure the robustness and credibility of the evaluation process, 3 highly qualified and experienced professors were carefully selected to assess the ChatGPT-generated answers. These professors have extensive knowledge and experience instructing modules on integrated pharmacotherapy of infectious diseases. Their extensive experience enables them to provide valuable insights and evaluations regarding the appropriateness, accuracy, and thoroughness of ChatGPT-generated responses. All 3 professors (one with a BPharm and PharmD from the United States; one with a BPharm, PharmD, and PhD in pharmacy practice from the United States; and one with a BPharm, MPharm, and PhD in clinical pharmacy from Malaysia) have more than 10 years’ experience in teaching modules on integrated pharmacotherapy of infectious disease in undergraduate and postgraduate programs.

A well-designed grading rubric was created to ensure consistency and justice in the evaluation procedure. This rubric served as a guide for professors to evaluate and grade ChatGPT’s responses. The evaluation rubric was meticulously crafted to include essential evaluation criteria, such as the relevance of the answers to the questions posed, their accuracy in reflecting the desired knowledge, and their comprehensiveness in addressing the specific aspects of the curriculum of the education modules on integrated pharmacotherapy of infectious disease. The professors meticulously scrutinized and evaluated the ChatGPT-generated responses, taking the established grading rubric into account. Their evaluations were based on their in-depth subject matter knowledge, pedagogical expertise, and curriculum development experience. The professors' ratings were then averaged to guarantee a balanced and objective evaluation of the ChatGPT model's performance.

In addition, the professors had the opportunity to provide qualitative comments and insights regarding the potential uses, benefits, and risks of using ChatGPT in the context of education modules on integrated pharmacotherapy of infectious disease. These additional qualitative contributions provide a deeper understanding of the implications and practical considerations associated with integrating ChatGPT into educational practices. Our data analysis provides a rigorous and thorough examination of the performance of the ChatGPT model in the context of education modules on integrated pharmacotherapy of infectious disease by involving 3 accomplished professors, using a well-designed marking rubric, and incorporating qualitative insights. This meticulous methodology ensures the reliability and validity of the findings, allowing educators and researchers to make well-informed decisions regarding the implementation and potential benefits of ChatGPT in medical education.

### Ethical Considerations

This project protocol was assessed and exempted for ethics approval by the Research Committee of the College of Medical Sciences, Azal University for Human Development (REC-2022-36).

## Results

### Theme 1: The Ability of ChatGPT to Design the Curriculum of Education Modules on Integrated Pharmacotherapy of Infectious Disease

#### Step 1: Problem Identification and General Needs Assessment

##### Overview

Our analysis of the experts’ opinions shows that ChatGPT was able to describe the need for the integrated pharmacotherapy curriculum in general for health care students and describe the issue of antibiotic resistance; however, it was unable to describe the importance of integrated pharmacotherapy of infectious disease. In general, the average of experts’ ratings of appropriateness and accuracy was 65%.

##### Potential Benefits

ChatGPT can help medical and health sciences educators by highlighting the importance of integrated pharmacotherapy curricula from reviewing the literature.

##### Potential Risks

ChatGPT could not describe the problem and carry out a general needs assessment for a specific population.

##### Recommendations

Medical and health sciences educators can use ChatGPT as a guide for understanding what is reported in the literature; then, they should be able to understand the problem and carry out a general needs assessment in the context of their countries with other methods.

#### Step 2: Targeted Needs Assessment

##### Overview

Our analysis of the experts’ opinions shows that ChatGPT was able to design a general initial questionnaire to use for the feasibility study of integrated pharmacotherapy; however, ChatGPT was unable to design a specific questionnaire related to integrated pharmacotherapy of infectious disease. Furthermore, ChatGPT was not able to design a qualitative study. The average of experts’ ratings of appropriateness and accuracy was 50%.

##### Potential Benefits

ChatGPT can help medical and health sciences educators to design a quick questionnaire to be used for conducting feasibility studies.

##### Potential Risks

There are many steps involved in designing valid and reliable questionnaires or qualitative interviews, which ChatGPT will not be able to undertake.

##### Recommendations

Medical and health sciences educators cannot use ChatGPT to develop valid and reliable questionnaires and qualitative interviews.

#### Step 3: Goals and Objectives

##### Overview

Our analysis of the experts’ opinions shows that ChatGPT could design the goals for the curriculum of the education modules on integrated pharmacotherapy of infectious disease, and the average of experts’ ratings of appropriateness and accuracy was 92%. ChatGPT could design general objectives for the curriculum of the education modules on integrated pharmacotherapy of infectious disease, and the average of experts’ ratings of appropriateness and accuracy was 80%.

##### Potential Benefits

ChatGPT can help medical and health sciences educators to design goals and objectives for the curriculum of the education modules on integrated pharmacotherapy of infectious disease.

##### Potential Risks

The goals and objectives suggested by ChatGPT were not specific and could not cover all learning objectives or outcome domains.

##### Recommendations

Medical and health sciences educators can use ChatGPT as a guide for preparing goals and objectives related to the curriculum of education modules on integrated pharmacotherapy of infectious disease.

#### Step 4: Educational Strategies

##### Overview

Our analysis of experts’ opinions shows that ChatGPT could help in the development of educational strategies, and the average of the experts’ ratings of appropriateness and accuracy was 75%.

##### Potential Benefits

ChatGPT can help medical and health sciences educators to develop educational strategies.

##### Potential Risks

The educational strategies suggested by ChatGPT could not be completed.

##### Recommendations

Medical and health sciences educators can use ChatGPT as a guide to develop educational strategies related to the curriculum of education modules on integrated pharmacotherapy of infectious disease.

#### Step 5: Evaluation and Feedback

Our analysis of experts’ opinions shows that ChatGPT could help suggest suitable evaluation and feedback, and the average of the experts’ ratings of appropriateness and accuracy was 85%.

##### Potential Benefits

ChatGPT can help medical and health sciences educators with teaching and learning evaluation and feedback methods (for different courses and programs).

##### Potential Risks

The suggested evaluation and feedback methods by ChatGPT could not be completed.

##### Recommendations

Medical and health sciences educators can use ChatGPT as a guide in the evaluation and feedback related to the curriculum of education modules on integrated pharmacotherapy of infectious disease.

### Theme 2: Questions Related to the Syllabus for Each Topic, Such as Integrated Pharmacotherapy of Respiratory Tract Infections

#### Overview

Our analysis of the experts’ opinions shows that ChatGPT could help in syllabus design, and the average of the experts’ ratings of appropriateness and accuracy was 70%. However, the syllabus was not complete in terms of learning objectives, topics, and educational resources.

#### Potential Benefits

ChatGPT can, with caution, help medical and health sciences educators to design lecture notes for the curriculum of education modules on integrated pharmacotherapy of infectious disease.

#### Potential Risks

The suggested lecture notes by ChatGPT could not be completed and missed many important issues.

#### Recommendations

Medical and health sciences educators can use ChatGPT as a guide in preparing the syllabus of the curriculum of integrated pharmacotherapy of infectious disease.

### Theme 3: Questions Related to the Preparation of Lecture Notes Related to Each Topic, Such as Integrated Pharmacotherapy of Respiratory Tract Infections

#### Overview

Our analysis of experts’ opinions shows that ChatGPT could help prepare lecture notes; however, the lecture notes were not complete, and the suggested learning objectives or outcomes for each lecture were not complete. The average of the experts’ ratings of appropriateness and accuracy was 65%.

#### Potential Benefits

ChatGPT can, with caution, help medical and health sciences educators to design the syllabus of the curriculum of integrated pharmacotherapy of infectious disease.

#### Potential Risks

The syllabus suggested by ChatGPT could not be completed and missed many important issues.

#### Recommendations

Medical and health sciences educators can use ChatGPT as a guide in preparing lecture notes for the curriculum of integrated pharmacotherapy of infectious disease.

### Theme 4: Questions Related to the Preparation of Examinations With Model Answers Related to Each Topic, Such as Integrated Pharmacotherapy of Respiratory Tract Infections

#### Overview

Our analysis of expert’s opinions shows that ChatGPT could help in preparing model answers for examinations. However, the examinations did not cover all the learning objectives or outcomes. The average of experts’ ratings of appropriateness and accuracy was 70%.

#### Potential Benefits

ChatGPT can, with caution, help medical and health sciences educators to prepare model answers for different types of examinations related to the curriculum of integrated pharmacotherapy of infectious disease.

#### Potential Risks

The examination questions suggested by ChatGPT could not be completed and did not cover the learning objectives or outcomes.

#### Recommendations

Medical and health sciences educators can use ChatGPT as a guide in preparing examinations for the curriculum of integrated pharmacotherapy of infectious disease.

## Discussion

### Background

This study explored the ability of ChatGPT to help medical and health sciences educators in curriculum design, syllabus design, lecture notes preparation, and examination preparation. The findings of this study can be classified into 3 themes.

### Theme 1: Potential Benefits of Using ChatGPT in the Curriculum of Integrated Pharmacotherapy of Infectious Disease

Our findings show that ChatGPT was able to help medical and health sciences educators, especially new educators, in all aspects of curriculum development with caution, and the experts rated the curriculum development aspects between 50% in the targeted needs assessment and 92% for suggestions about goals. Therefore, medical and health sciences educators can use ChatGPT as a guide in developing such a curriculum. ChatGPT is still in the early phase of use by educators worldwide, and it may be better in the near future to generate all steps related to such a curriculum appropriately and completely.

### Theme 2: Potential Risks of Using ChatGPT in the Curriculum of Integrated Pharmacotherapy of Infectious Disease

Our findings show that there are potential risks associated with using ChatGPT in the development of the curriculum of integrated pharmacotherapy of infectious disease, syllabus design, lecture notes preparation, and examination preparation, such as missing important learning objectives or outcomes, various examination questions, and others. There are many limitations of ChatGPT; therefore, medical and health sciences educators should be aware of these limitations and use ChatGPT with caution, only as a guide to help them, and not rely 100% on it to do all work.

### Theme 3: Recommendations for Using ChatGPT in the Curriculum of Integrated Pharmacotherapy of Infectious Disease

ChatGPT can help medical and health sciences educators in many ways, and they can use ChatGPT as a guide in curriculum design, syllabus design, lecture notes preparation, and examination preparation.

### Limitations

A limitation of our study is that our methodology could benefit from additional clarification and elucidation, particularly in regard to the rating process and performance evaluation. Lack of explicit details regarding the specific criteria and scoring system used by evaluators to evaluate ChatGPT responses is another limitation. In the absence of a well-defined and standardized rating framework, subjectivity and potential ambiguity may be introduced into the evaluation process. This could impact the results' dependability and comparability.

Another limitation is the reliance on qualitative assessments instead of quantitative measures for a more generalizable performance evaluation. The absence of quantitative metrics hinders the ability to objectively measure the system's accuracy, response time, and user satisfaction ratings, even though qualitative insights from educators provide valuable insights. Consequently, our findings may have limited applicability.

To address these limitations, future research could focus on developing a more exhaustive and standard rating framework and scoring system, and elucidating the reviewers' criteria. Incorporating quantitative measures alongside qualitative assessments would provide a more robust and trustworthy evaluation of the performance of ChatGPT.

### Conclusions

This study highlights the immense potential of ChatGPT as a valuable tool for medical and health sciences educators in various aspects of the curriculum of integrated pharmacotherapy of infectious disease. The findings emphasize both the benefits and risks of incorporating ChatGPT into educational practices, providing valuable insights for educators seeking to leverage AI technology to improve teaching and learning. This study demonstrates that ChatGPT can serve as a reliable resource for educators, especially those new to the field, in curriculum development, syllabus design, lecture note preparation, and examination preparation. Educators should exercise caution and use ChatGPT as a supplementary resource, rather than relying solely on its outputs, in order to ensure its effective and responsible use. Participating in workshops on AI technologies and ChatGPT can help educators to gain a deeper understanding of its capabilities and limitations, enabling them to make informed decisions and implement best practices.

## Abbreviations

AI: artificial intelligence
LLM: large language model
